# THE BUILT ENVIRONMENT INFLUENCED THE INCIDENCE OF FALLS IN OLDER ADULTS LIVING IN THE UNDERSERVED COMMUNITY

**DOI:** 10.1093/geroni/igz038.1076

**Published:** 2019-11-08

**Authors:** Rie Suzuki, Jennifer Blackwood, Shailee Shah, Sabah Ganai, Kimberly Warden

**Affiliations:** 1 Department of Public Health and Health Sciences, University of Michigan-Flint, Flint, Michigan, United States; 2 Physical Therapy Department, University of Michigan- Flint, Flint, Michigan, United States; 3 Hamilton Community Health Network, Inc., Flint, Michigan, United States

## Abstract

The built environment is commonly cited as a facilitator to local walking. Although health promotion programs targeting physical activity are available, few studies have investigated the associations of the perceived neighborhoods with the incidence of falls in the minority communities. Hence, the purpose of this preliminary study was to understand whether the perceived built environment influenced the fall experiences in older adults living in the underserved community. The preliminary cross-sectional survey was conducted at the regional health clinic in Flint, MI. Descriptive statistics and analysis of variance (ANOVA) were performed using SAS v8.4. The eligibility criteria included over 65 years old and Flint residents. Of 132 participants, the mean age was 69.75 (SD=5.00). The majority were female (68%), African Americans (80%), single, divorced or widowed (80%), and > GED (84%). The ANOVAs supported that “had fallen in the past year” was associated with “stores are within easy walking distance,” “easy to walk to a transit stop” and “there is a dirt strip that separates the streets from the sidewalks.” The fall experience was more likely to associate with the sedentary lifestyle and the comorbidities such as diabetes, fatigues, muscle spasms, and chronic pain. To summarize, the built environment increased the incidence of falls in the past year. Those who had fallen had poor health conditions. Further studies are needed for older adults to engage in physical activity. It is essential to develop the age-friendly support systems and accommodations to local walking in this community.

